# Outcomes of internal fixation for pediatric proximal femoral fractures using a 3.5 mm T-plate

**DOI:** 10.1007/s00402-026-06193-8

**Published:** 2026-03-06

**Authors:** Mohamed I. Abulsoud, Mohamed G. Hussiny, Samir A. Nematallah, Mohammed Al Nahhas, Ibrahim M. Elsebaey, Emad Zayed, Mohamed F. Elhalawany, Mostafa M. Elgahel, Yahia A . Hassanein, Elsayed Shaheen, Mohamed H. Abdou, Mahmoud M. Hassan

**Affiliations:** 1 Department of Orthopaedic Surgery, Faculty of Medicine., Al-Azhar University, Cairo, Egypt; 2 Division of Orthopaedics, Department of Surgery, Faculty of Medicine, Jazan University, Jazan, Saudi Arabia

**Keywords:** Proximal femoral fractures in children, Paediatric femoral neck fractures, Pediatric fractures, Fracture neck femur, Delbet classification.

## Abstract

**Objective:**

This study aims to investigate the functional and radiographic outcomes after internal fixation of proximal femoral fractures in children using a 3.5 mm T-plate.

**Methods:**

From December 2015 to September 2022, all cases presenting with recent proximal femoral fractures were treated with internal fixation using a 3.5 mm T-plate. All patients were followed up for 24 months. Union time, neck shaft angle, neck length, quality of reduction, and functional outcomes were assessed.

**Results:**

The study included 37 patients; the mean age was 8.6+/- 1.6 (6–11) years. Delbet type IV was the most frequent fracture type (13 cases, 35.1%), followed by type V (10 cases, 27%), and type III (9 cases, 24.3%), with type II accounting for 5 cases. All fractures achieved union with a mean time of 8,7 weeks (range 6–12 weeks), As regards radiographic parameters, the mean neck shaft angle difference was 4.9 (SD 3.7) degrees (range 0–16 degrees) less than the contralateral side, the mean neck resorption ratio was 93.8 +/- 4 (range 85–100), The quality of reduction was anatomical in 24 cases (64.9%), acceptable in 10 cases (37%), and considered unacceptable in 3 cases (8.1%), The functional outcome was good in 24 cases (64.9%), fair in 11 patients (29.7%) and poor in two patients (5.4%).

**Conclusion:**

The use of a contoured 3.5 mm conventional T-plate in the fixation of recent pediatric femoral fractures (types II–V) yields good results regarding union rates, functional outcome, and maintaining the mechanics of the hip.

## Introduction

Proximal femoral fractures are rare injuries in the skeletally immature population, with an incidence of about 0.45 per 100,000 children aged less than 16 years. And constitute about 1% of all fractures in this age group. Males are more predominant than females (1.5-2:1), most frequently at the age of nine. Most cases occur due to road traffic accidents and falls from heights [1–4].

The most widely used classification system for proximal femoral fractures is the Delbet classification, which Colonna popularized about a century ago. Type I fractures are trans-physeal, Type II are transcervical, Type III are basal-trochanteric, and Type IV are intertrochanteric fractures. Type II Delbet fractures are the most common, followed by types III and IV, and the least common are type I fractures [2]. Azouz et al. [5] added type V fractures, which are subtrochanteric; the more proximal the fracture (type I), the higher the incidence of AVN [6].

Proximal femoral fractures are prone to high complication rates, especially avascular necrosis (AVN) of the femoral head, which accounts for about 29% of cases [7]. It is usually presented around 9–13 months after injury; about 59% of cases are symptomatic, and 65% progress to the collapsed stage [8], Initial displacement, location (type) of the fracture, and older age group are the most predictive factors for the development of AVN [9, 10]. Avascular necrosis of the femoral head can be classified according to Ratliff’s classification into type 1, which is diffuse with total head collapse; type 2, which is segmental involvement of the femoral head with minimal head collapse; and type 3, which is metaphyseal changes that are confined to the femoral neck. Types 1 and 2 are associated with poor prognosis [11, 12].

The treatment of proximal femoral fractures is surgical to avoid the consequences of poor outcomes like AVN, non-union, or coxa vara. Non-operative treatment could be considered at very young ages, less than 2 years old, with incomplete fractures or severely ill children; loss of reduction, even in reduced complete fractures, is quite common [2, 7, 13].

Several options for fixation exist in orthopaedic practice, depending on the age, type of fracture, degree of comminution, and availability of implants. The most popular contemporary types of implants include K-wires, cannulated cancellous screws, pediatric Dynamic hip screws, and fixed-angle locked plates [2, 3, 14].

This study aims to investigate the functional and radiographic outcomes after internal fixation of proximal femoral fractures in children using a 3.5 mm T-plate.

The research question is: What are the functional outcomes and complication rates if a 3.5 mm T-plate has been used in the fixation of paediatric proximal femoral fractures?

The study hypothesizes that a small fragment T-plate is an effective tool in managing such fractures in this age group.

## Patients and methods

This prospective research comprised thirty-seven consecutive patients from December 2015 to September 2022.

The Institutional Ethical Committee approved the study. All methods in this study were conducted in accordance with the institutional research committee’s ethical standards, the 1964 Helsinki Declaration and its subsequent revisions, or similar ethical standards. According to the guidelines of the hospital’s research ethics committee, informed consent was obtained from the guardians of all individual participants included in the study.

The STROBE (Strengthening the Reporting of Observational Studies in Epidemiology) principles were followed for cohort studies.

Inclusion criteria included children from 6 to 12 years old at the time of injury, who presented with isolated, traumatic, recent (within 24 h), proximal femoral fractures, Delbet type II, III, IV [2], and Azouz type V [5], which were treated by internal fixation with a conventional 3.5 mm T-plate.

Exclusion criteria included Delbet type I fractures, pathological fractures, stress fractures, open fractures, adolescents, associated lower limb fractures, cases with delayed presentation of more than 24 h, pre-existing ipsilateral or contralateral old hip pathology, and polytrauma patients.

### Surgical technique

All cases were performed under general anaesthesia after thorough clinical, laboratory, and radiological examination to assess their fitness for surgery. Written consent for surgery was obtained from the parents or guardians of the patients. A senior surgeon, familiar with the use of the plate and with a minimum of ten years of experience in orthopaedic trauma, served as the primary surgeon. All surgeries were performed within 24 h after injury.

A first-generation cephalosporin was administered with the induction of anesthesia in a dose of 100 mg/Kg, with a maximum of 2 g as a single dose.

**Fig. 1:**
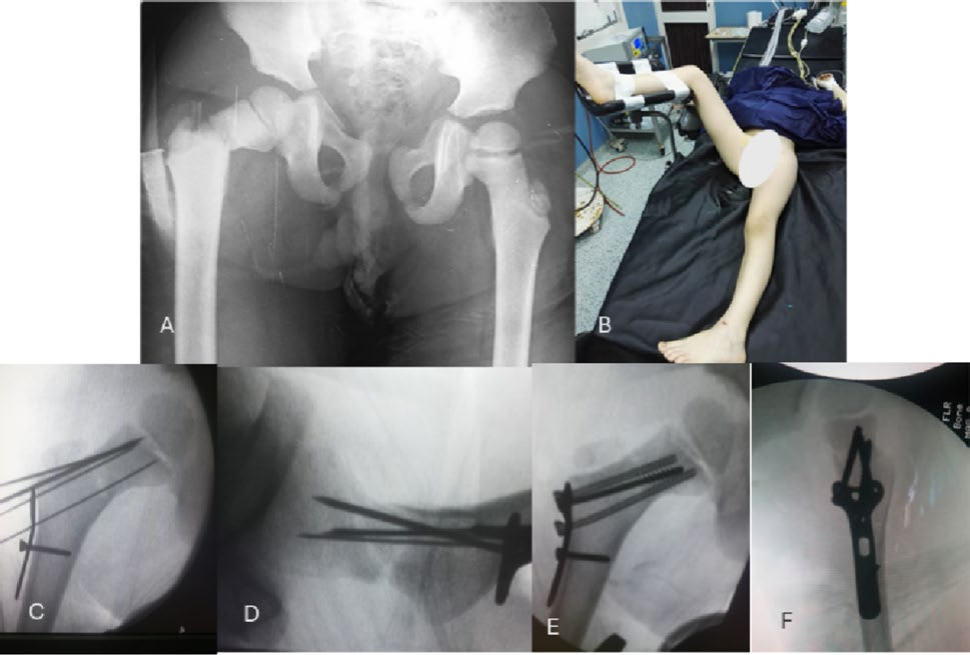
**A** Anteroposterior X-ray view of both hip joints showing a Delbet type II proximal femoral fracture. **B** The positioning of the patient on the operating radiolucent table. **C** and **D** show Intraoperative Fluoroscopic images showing the preliminary reduction, which k-wires have maintained, and the plate has been applied with a screw in the adjustment hole in the plate. Cannulated guidewires could be used for preliminary fixation, which could be replaced by cancellous screws 4 mm. **E** and **F** Fluoroscopic photos after plate fixation to the proximal fragment in anteroposterior and axial views

All cases were positioned in the supine position on a radiolucent table. The contralateral limb has been rested on a foot piece after protection of the bony prominences to enable obtaining lateral fluoroscopic views without the need to change the limb’s position during surgery, as this may alter the maintenance of the reduction (Fig. 1).

The proximal femur was exposed via the standard lateral approach. Capsulotomy was not performed routinely, but only when an open reduction was necessary in cases of Delbet types II or III. Open reduction was performed after three trials of closed reduction failed to achieve anatomical reduction according to Song’s criteria [15], i.e., no displacement or angulation in both planes.

The reduction was maintained with the use of preliminary K-wires. Then, the 3.5 mm T-plate was used, The horizontal part of the plate was contoured to match the NSA of the patient, which enables the surgeon to make the screws parallel to the long axis of the neck, in this age group, NSAs are highly variable so they should be contoured for every patient, in cases of subtrochanteric fracture, it is easier to pre-contour the plate because the NSA is already usually preserved.

A guidewire was used to ensure that the central hole in the horizontal end of the plate allows the central positioning of the screw in the femoral neck in all planes.

Partially threaded 4 mm cancellous screws have been used in type II and III fractures to achieve compression of the fracture as much as possible without the need to penetrate the growth plate of the femoral head (Fig. 1).

Fracture fixation was concluded by inserting 3–4 cancellous screws 4 mm proximally and three cortical 3.5 mm screws distally according to fracture type and the age of the patient. A final fluoroscopy was taken to ensure the quality of reduction and fixation.

A hip spica was used to protect patients younger than seven years old; for those older than 7 years, a hip abduction brace was used instead. Spica or brace is discontinued after 4–6 weeks, guided by follow-up X–rays.

All cases were kept non-weight-bearing for 6 weeks. Partial weight-bearing as compliant by children and their guardians, progressing the weight-bearing and activity of daily living guided by the pain, and their ability to ambulate.

The guardians were instructed on the schedule of immobilization, weight-bearing, and the time for return to activity (including school and sports activities), as well as the need for plate removal 12–24 months postoperatively.

**Fig. 2:**
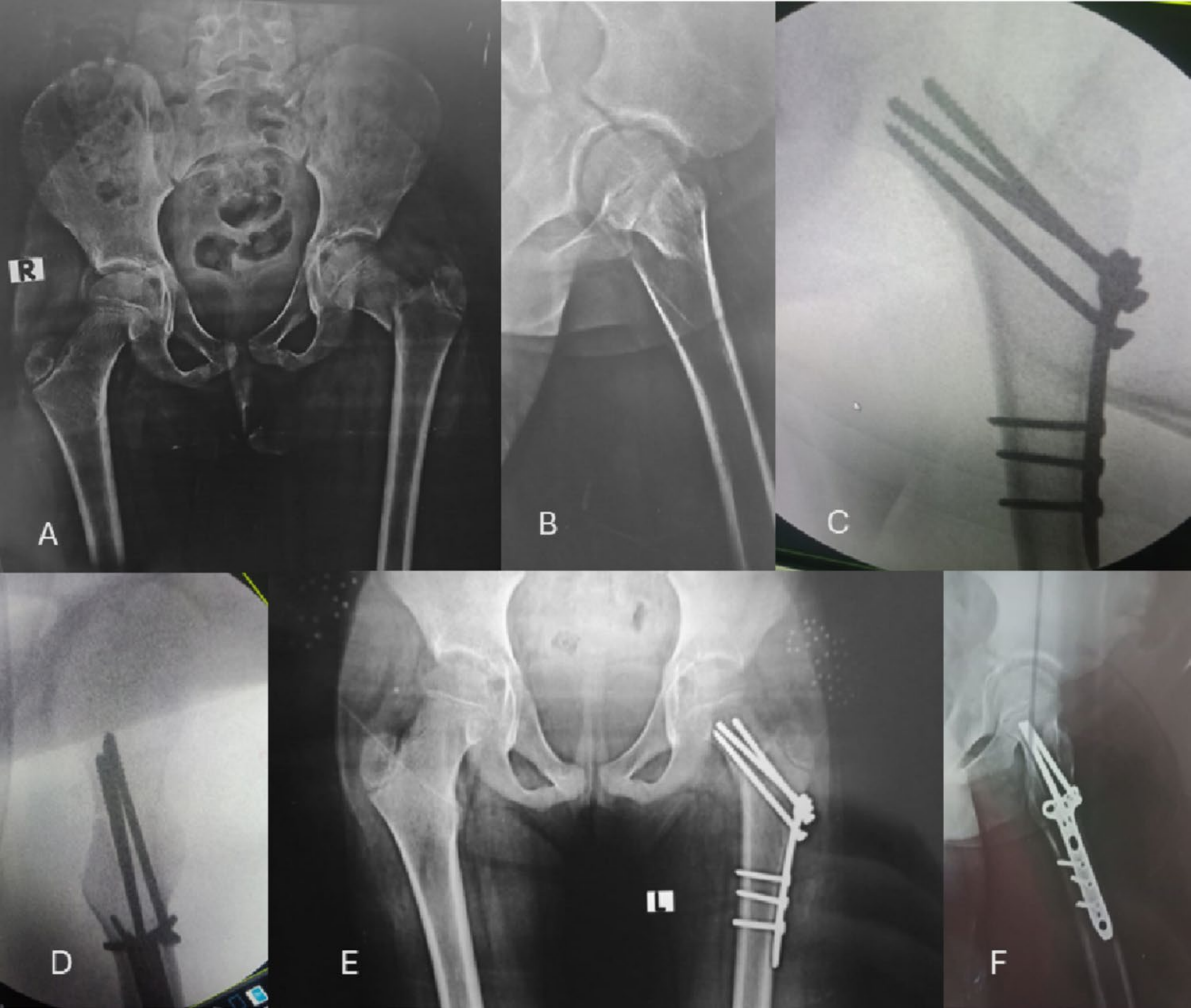
**A** and **B** show an X-ray anteroposterior view of both hip joints and a lateral view of the left hip joint showing a Delbet type III proximal femoral fracture. **C** and **D** show Intraoperative Fluoroscopic images showing intraoperative reduction and fixation using a contoured 3.5 mm T-plate. **E** and **F** Follow-up X-ray 6 months postoperatively showing the maintained hip radiographic parameters with healing of the fracture

**Fig. 3:**
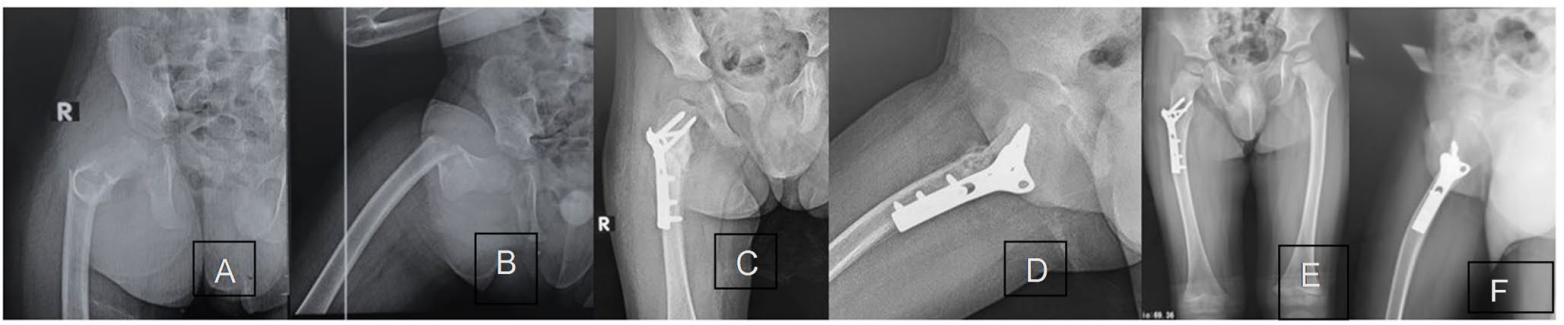
**A** and **B** show X-ray anteroposterior and a lateral view of the right hip joint showing a Type V proximal femoral fracture. **C** and **D** show Follow-up rays 4 weeks postoperatively, showing the reduction and fixation of the fracture with callus formation at the fracture site. **E** and **F** Follow-up X-ray 20 months postoperatively before implant removal, showing the maintained hip radiographic parameters with the normal growth of the proximal femur as compared to the contralateral side

Figures 2 and 3 are case presentations.

### Outcome measures

All patients were subjected to follow–up for 24 months by scheduled visits. They were assessed for wound care until the removal of stitches, which occurred within 10–14 days postoperatively, and then for radiological and functional outcomes.

The following radiological parameters were measured: fracture union time, quality of reduction, neck-shaft angle, and neck resorption ratio (NRR). Functional outcome was also evaluated after a 24-month follow-up.

Coxa vara was defined as the neck-shaft angle being less than 120 degrees or more than 10 degrees when compared to the contralateral side [3, 16]. The neck resorption ratio (NRR) expressed the length of the neck in comparison to the contralateral side [17].

The quality of reduction was stratified according to the criteria of Song [15] as anatomically acceptable or unacceptable.

No displacement or angular deformity characterizes anatomical reduction; acceptable reduction is defined as displacement of less than 2 mm or angular deformity within 20° of the normal neck shaft angle on anteroposterior and axial radiographs; and unacceptable reduction is defined as displacement greater than 2 mm or angular deformity greater than 20° on anteroposterior or axial radiographs.

The functional outcome was assessed 24 months postoperatively using the Ratliff system [18], which classified cases into good, fair, or poor outcomes. A paediatric orthopaedic consultant trained the assessors in using the functional outcome score.

Radiographic analysis was performed by two authors who did not participate in the surgical work to minimize bias. Both authors have at least 10 years of experience in general orthopaedic surgery and are familiar with measuring the required parameters. They have been explained as a revision of the principles before starting the real work; a senior author revised non-identical measures.

## Results

The study included 40 patients, three patients discontinued follow up due to travelling with their family, remained 37 patients who completed the whole follow up period (24 months ), the mean age was 8.6+/- 1.6 (6–11) years, twenty-two of them (59.5%) were males, the right hip was involved in twenty of them (54.1%), the mechanism of injury in 21 of the cases (56.75%) was a road traffic accident, 8 cases presented after a fall while playing, 5 cases had fallen downstairs, and 3 cases after domestic fall.

As regards the fracture types, Delbet type IV was the most frequent fracture type (13 cases, 35.1%), followed by type V (10 cases, 27%) and type III (9 cases, 24.3%), followed by type II, where five cases were included (13.5%) (Table 1).

**Table 1:** Demographic data

Parameter	Data
*Age (years)*	
Mean ± SD. (range)	8.6 ± 1.6
	(6–11)
*Gender*	
Male	22 (59.5%)
Female	15 (40.5%)
*Side*	
Right Left	20 (54.1%) 17(45.9%)
*Mechanism of injury*	
Road traffic accident Fell while playing Falling downstairs Domestic fall	21 (56.75%) 8 (21.62%) 5 (13.51%) 3 (8.1%)
*Fracture type*	
Type II Type III Type IV Type V	5 (13.51%) 9 (24.32%) 13 (35.13%) 10 (27.02%)

All fractures achieved union with a mean time of 8.7 (SD1.9) weeks (range 6–12 weeks), Union has been defined clinically by subsiding of pain while bearing weight, and radiographically by disappearance of pre-existing fracture line, As regards radiographic parameters, the mean neck shaft angle difference was 4.9 (SD 3.7) degrees (range 0–16 degrees) less than the contralateral side, coxa vara was observed in 2 cases (5.4%), the mean neck resorption ratio as a parameter to measure shortening of the femoral neck was 93.8 (SD 4) (range 85–100) (Table 2).

**Table 2:** Results of the union time and radiographic parameters

Parameter	Minimum	Maximum	Mean	SD
union time (weeks)	6	12	8.76	1.86
NRR	85	100	93.78	4.04
NSA (degrees)	0	16	4.94	3.69

The quality reduction was anatomical in 24 cases (64.9%), acceptable in 10 cases (27%), and considered unacceptable in 3 cases (8.1%). In the three cases of unacceptable reduction, there was angulation in the frontal plane (varus more than 20 degrees in one case, and two cases with angulation more than 20 degrees in the axial plane); there were no cases of displacement greater than 2 mm.

The functional outcome of the patients at the end of the follow-up, after 24 months, was good in 24 cases (64.9%), fair in 11 patients (29.7%), and poor in two patients (5.4%).

Regarding complications, Avascular necrosis was observed in 5 cases (13.5%), with three cases being Delbet type III. Two cases were Delbet type II, wound infection in two patients (5.4%) which was treated by antibiotics and serial wound care and improved without the need for surgical debridement, difficulty in screw removal was observed in 5 cases (13.5%), all of them was in the posterior screw in the proximal part of the plate, so the authors decided not to use this hole in following cases, hence that time, no significant difficulties in hardware removal were detected (Table 3).

**Table 3:** The results of the functional outcome, quality of reduction, and complications

Variable		Frequency		Per cent
*Functional outcome*				
Good		24		64.9
Fair		11		29.7
Poor		2		5.4
Total		37		100
*Quality of reduction*				
Anatomical		24		64.9
Acceptable		10		27.0
Unacceptable		3		8.1
*Complications*				
AVN		5		13.5
Infection		2		5.4
Screw removal difficulties		5		13.5

No cases were detected for growth plate arrest or remarkable symptomatic leg length discrepancy.

## Discussion

Proximal femoral fractures in children are uncommon injuries that could have an impact on the patient’s quality of life for a long time. The study demonstrates that the use of a small fragment T-plate in the fixation of such fractures results in an excellent union rate, preserving the neck-shaft angle, and does not disturb the growth of the proximal femur, yielding acceptable functional outcomes in approximately 95% of cases.

The fixation device should withstand the forces around the hip until the fracture union occurs [19]. The literature discussed several points of debate regarding pediatric proximal femoral fractures [2, 3, 6]. However, the fixation method is still under discussion, as it depends on factors such as age, fracture type, degree of comminution, implant availability, and the surgeon’s preferences.

Screws were commonly used in Delbet type II and III fractures. Biomechanically, the use of plate constructs is superior to cancellous screws in the fixation of femoral neck fractures in adults [20, 21]; the use of screws in children is associated with an increased rate of coxa vara [15].

In the study by Wang et al. [22], which included 239 patients, the rate of AVN in patients treated with open reduction and screw and plate fixation was significantly lower than that in those treated with cannulated screws or K-wires. The same findings were reported by Spence et al. [5].

The use of the Locking Compression Pediatric Hip Plate is another appealing option, yielding excellent outcomes in cases of proximal femoral osteotomies and tumors [23–26]. The use of such plates is not commonly reported in the literature for fractures, perhaps due to the possibility of catastrophic failure, which has been reported in young adults [27]. However, a recent study by Zhou et al. [28] presents contradictory results, as proximal femoral locking plates in adults were associated with shorter union times, better maintenance of neck length, and improved functional outcomes compared to cancellous screws; both options had a similar complication rate.

Recently, Cope et al. [29] concluded that the early failure rate of proximal femoral locked plates was 15.4% (4 out of twenty-six cases), while there was no failure rate in other methods of fixation. Other explanations are the availability of simpler, less technically demanding, and more economical implants. In a recent study, Haider et al. [30] compared the outcomes of Proximal femoral locked plates with cannulated screws in the management of 42 patients presenting with Delbet type II and III fractures. The study found no difference between the two groups except for the articulo-trochanteric distance as an indicator for proximal femoral alignment.

The proximal humeral locked plates have been described in the treatment of subtrochanteric fractures [31, 32]; however, the screw distribution of the plate and its size could alter its use in other fracture types.

The study included Azouz type V [5] fractures because the authors believe that they could have the same treatment options; the AO classification added subtrochanteric fractures as a subtype of pediatric proximal femoral fracture [33]. While type I fractures have been excluded from the study because smooth K-wires are safer in treating them to preserve the physis [3]. The age group (6–12 years old) has been determined because this age group shares the exact incidence, morphology, and blood supply of the femoral head [2].

The timing of surgery in proximal femoral fractures in children is still a matter of debate; some studies found that timing does not appear to be an essential risk factor for AVN [7, 8], while some studies found that earlier presentation is associated with less complication rate including AVN and changes in hip geometry [34], as a measure to reduce bias, the study included patients which were presented within twenty-four hours post injuries.

Current evidence suggests that there is no difference in outcome or the incidence of complications between closed and open reduction in cases of femoral neck fractures, provided anatomical reduction can be achieved [15]. Cases with closed or open reduction were included in the study.

The results of the study are comparable to what exists in the literature; the incidence of coxa vara after pediatric hip fractures was reported as 18% when all types of hip fractures were investigated together [35], and the difference of greater than 10° in the neck-shaft angles between the two hips became clinically significant [16]. In this study, the mean neck shaft angle was 4.9 degrees compared to the contralateral side; coxa vara was found in only two cases (5.4%).

According to the systematic review of Pandey and John [6], the functional outcome when measured According to Ratliff’s criteria, 62.54% (339 out of 542) patients had good, 19.5% fair and 18.4% poor functional outcomes, respectively, which is comparable to the results of this study (good in 64.9%of cases, fair in 29.7% and poor in 5.4% of cases which represent two patients).

In the study by Yerli et al. [36], which retrospectively analyzed 35 patients, 15 patients (42.9%) had a type 2 Delbet fracture, five patients (14.2%) had a Delbet type 3 fracture, and 15 patients (42.9%) had a Delbet type 4 fracture. AVN was seen in four (11.4%) patients. Additionally, coxa vara was observed in six (17.2%) patients.

In this study, the incidence of fracture type was different than what is already reported in the literature [2, 4, 36]. Delbet type IV was the most prevalent fracture type (35.1%), followed by type V (27%) and type III (24.3%), followed by type II (13.5%). This could be due to a change in the mechanism of injury (56.75% due to road traffic accident), while in Regmi et al. [34], 52.27% of cases were due to a fall from height. In the study of Mirdad on 14 patients [4], Eight cases (57.1%) were due to road traffic accident and 6 (42.9%) were due to falling from height, or due to adding type V to the sample size, also the reader should take the incidence of AVN in this study (13.5%) in the light that there were 62% of cases type IV and V fractures which are low risk of AVN than other types.

In Delbet type II fractures, the proximal fragment is typically short, and the available 4.00 mm partially threaded screws are provided with fixed thread diameter, so compression of the fracture using those screws is sometimes not possible. Anatomical reduction in such a situation is crucial, and other methods of compression (reduction forceps) are very important.

Most authors recommend elective implant removal between 9 and 22 months postoperatively [6]. The removal in most cases was uneventful, except for the posterior screw of the proximal fragment in 5 cases, where difficulties ranged from being bent due to overstress to being found with a broken head. Specific instruments were used for extraction from the screw removal set. So, it is not recommended to use such a screw; three other screws in the proximal fragment were sufficient to maintain the reduction with a stable construct.

### Limitations

Although the study shows that available and economic implants give good outcomes in most proximal femoral fractures, the study carries some limitations: -.

The lack of comparison with other fixation options, being a single-center study, and the relatively small cohort are potential limitations. Additionally, the absence of biomechanical studies is due to the unique nature of the pediatric femur, which cannot be reproduced in the same manner as the adult femur.

## Conclusion

The use of a contoured 3.5 mm conventional T-plate in the fixation of recent pediatric femoral fractures (types II–V) yields good results regarding union rates, functional outcome, and maintaining the mechanics of the hip.

## Data Availability

No datasets were generated or analysed during the current study.
